# Individuals experiencing disability and the ableist physical literacy narrative: critical considerations and recommendations for practice

**DOI:** 10.3389/fspor.2023.1171290

**Published:** 2023-10-09

**Authors:** Kyle Pushkarenko, Elizabeth Howse, Nicholas Gosse

**Affiliations:** School of Human Kinetics and Recreation, Memorial University of Newfoundland, St. John’s, NL, Canada

**Keywords:** critical analysis, physical literacy, disability, inclusion, ableism, practical recommendations

## Abstract

Physical literacy (PL) has been readily accepted and integrated globally, including organizations affording services to individuals experiencing disability. Despite its uptake, recent research has illustrated that understandings of PL reflect the normative standards of those who do not experience disability, leading to practices that diminish the unique and embodied capability of others while simultaneously validating ableism. While a shift towards recognizing and valuing the heterogeneity associated with PL has recently occurred, the ableist narrative persists. As a result, the operationalization of PL directly contradicts its conceptualization, fostering a physical activity climate that continues to marginalize individuals experiencing disability. With this in mind, this paper critically unpacks PL, challenging the existing ableist narrative and offering suggestions to heighten the level of inclusivity that underscores PL. Pathways, where physical activity professionals contribute to reproducing ableism, will be discussed.

## Introduction

Posited as a means of increasing physical activity and its subsequent positive health outcomes over the lifespan (1, 2), the concept of physical literacy (PL) has been enthusiastically integrated into various fields such as sport, recreation, and public health, becoming an item of interest for the development of organizational and national initiatives, policies and practices (3, 4). Defined as “the motivation, confidence, physical competence, knowledge and understanding to value and take responsibility for engagement in physical activities for life” (5),^1^ PL is framed according to one's embodied potential developed via interactions with the outside world (8). PL encompasses the skills, attitudes and lifestyle habits needed to enable active participation in movement cultures across an individual's lifespan (9, 10), and as such, has been positioned as essential to the overall development and enhanced quality of life of all individuals (6, 11, 12).

Grounded in notions of holism, embodiment, and lived experience (13), PL by its very nature, is proclaimed to be inclusive (14, 15). On this note, PL has served as a cornerstone for programming endeavors aimed at fostering sustained engagement in physical activities and fostering healthy, active lifestyles for all, including for individuals experiencing disability^2^ (17). Despite the efforts of initiatives (18–20) and organizations (21, 22) that have embraced the philosophical foundations of PL, and thus have committed to providing opportunities for everyone that are inclusive by design (i.e., those considered as “PL programs” rather than integrating PL into physical activity programs), the broader landscape of programming centered around PL development (i.e., physical activity programs integrating PL) continues to perpetuate the dominant narrative of PL.^3^ Consequently, these endeavours become entrenched in both overt and subtle expressions of ableism, failing to recognize the inherent value of PL irrespective of individual capabilities (21, 22). Additionally, they hinder attempts to comprehensively grasp and address inclusion from a socio-critical perspective (26–28). This leads to outcomes that devalue the embodied worth of individuals with disabilities, (re)construct an environment of physical activity that marginalizes and excludes (29), and (re)assert the primacy of the norm, consequently limiting the potential for appreciating individual differences (30).

While the subsequent position paper does not aim to pass judgment on physical activity programs employing PL as a means to ongoing physical activity engagement, its purpose is to initiate a thought-provoking discourse and contemplation about our utilization of PL to date. In fact, any resistance to claims made within this manuscript, is a step in the right direction given the importance of the topic itself and the dearth of discussion on it. Concurrently, this paper seeks to introduce novel approaches that uphold the foundational principles of inclusivity inherent to PL. It is our hope that such a discourse will not only shed some light on a relatively unexplored aspect of PL (i.e., inclusion in the context of disability) but also generate dialogue on how we can establish equitable physical activity climates and grant accessible opportunities (31) that fulfill the needs and desires of everyone, regardless of ability level.

## What is ableism?

Ableism, as defined by Campbell (32), encompasses a complex network of beliefs, processes, and practices that shape our understanding of self, body, and what it means to be fully human. It constructs a corporeal standard, projecting an idealized image of a “perfect, species-typical” (44) body and mind, demoting disability to a diminished state of being. Ableism operates as a supremacist ideology (33), establishing a socially constructed hierarchy of human worth. This ideology infiltrates our institutions and attitudes, influencing our appraisal of individuals and perpetuating the devaluation of those who do not conform to a sense of social normativity (32, 34, 35). Consequently, these individuals are socially constructed as Others—categorically different, naturally inferior, undesirable, and fundamentally requiring repair or modification (32, 33, 36).

As per Wolbring (36), “ableism is an umbrella ism for other isms such as racism, sexism, casteism, ageism …” (253). This phenomenon—ableism—stems from the preference for specific abilities within a social cohort, giving rise to hierarchies of privileges and discriminatory practices (36, 37). To illustrate, ableism manifests in tandem with racism when certain racial groups are attributed inherent qualities (e.g., cognitive prowess) that bestow them with societal advantages, leading to particular roles and responsibilities (38). Similarly, ableism emerges from ageism, where value and preference are linked to certain levels of capability, thus leading to biases and prejudiced treatment directed both at the younger members [referred to as “childism”; (39, 40)] and the elderly (41). Regardless, although this manuscript addresses ableism concerning (dis)ability, it is important to acknowledge that the concept has also been invoked by various social groups to legitimize their higher rights and elevated status in comparison to other groups (36, 37), a point worth mentioning. Encompassing the beliefs, processes and actions that put the value based on ability into practice (34), ableism occurs “in both *what* we do and *how* we go about doing it” (33). It subsists within institutional policies and practices that privilege and prefer able-bodiedness, reproducing unequal outcomes for disabled people to the benefit of able-bodied people [i.e., structural ableism; (42)]. Ableism is also reflected in the attitudes and expressions of bias between people, including those experiencing disability [i.e., interpersonal ableism; (43)], and occurs when individuals experiencing disability internalize society's prejudices, thereby perceiving themselves as inferior [i.e., internalized ableism; (32)]. These actions demonstrate and perpetuate a sense of “compulsory able-bodiedness” (44), arising from a contemporary social law dictating that individuals should strive to achieve and maintain the able-bodied ideal (i.e., physically fit, healthy, and non-disabled). Furthermore, they reinforce a societal attitude that uncritically asserts certain abilities as being inherently superior over others (45).

Despite well-meaning intentions of those attempting to foster an inclusive context for all, ableist assumptions persist within various disciplines and fields, including those devoted to providing physical activity opportunities for individuals experiencing disability. As such, it is challenging to identify and detach ourselves from these assumptions because they often form the unexamined foundations of our knowledge, practices, and values (46). This mystery is at the core of Lyons' (46) concept of “enlightened ableism”, whereby support for disability inclusion is masked by a level of uncertainty about ways to transform it into practice, resulting in the continuation of ableist practices and the inadvertent marginalization of people with disabilities (33).

## Reproducing ableism within PL practice

Despite the generation of newfound PL programs that embody the philosophy of the concept [i.e. (18–20)], the rapid uptake of PL has left it relatively misunderstood (12, 30). Ongoing debate associated with its defining features and meaning, coupled with a lack of empirical evidence supporting the concept's application (6, 23, 24), has generated substantial confusion and ambiguity amongst physical activity practitioners, rendering efforts to facilitate PL development inconsistent and incongruent (3, 47). This unevenness is exacerbated for individuals experiencing disability as ableist and normalized standards of individuals with privileged capabilities (i.e., individuals who do not experience disability) are often reproduced through practice (29, 48), likely in unintentional ways [i.e., enlightened ableism; (46)]. Such means of facilitation place individuals experiencing disability at a significant disadvantage regarding their PL development; a disadvantage existing prior to the onset of their so-called individualized journey (49), and one amplified by their constant and consistent engagement in physical activity programs not adhering to specific to need or capability (50). Not only do these practices devalue the difference existing with varied forms of embodiment, but they also run counter to the foundation of inclusion housed within the concept's underlying philosophy (5, 15), perpetuating a socially unjust system for individuals experiencing disability (29, 47).

One approach to PL development that can be considered ableist in practice (i.e., structural ableism) is a reliance on, and prioritization of, using physical development through physical activity programming as a means to capturing the essence of the concept as a whole. While it cannot be refuted that physical development (i.e., development of movement skills) is integral to the development of PL as a holistic concept (1, 51, 52), developmental opportunities emphasizing physical prowess [i.e., skill proficiency, physical competency and performance; (53)] can prove difficult or unrealistic as the requirements for participation and engagement supersede ability (28). Often, this results in the individual having a negative experience and, subsequently, a reduced willingness to return to engagement. Emphasizing physical development over aspects such as social engagement and enjoyment is thought to reinforce a particular capability or privilege and can lead to substantial and negative impacts on behalf of the participant [e.g., social exclusion, harm; (29, 48, 50)]. Additionally, physical activity practices used to facilitate PL development, and those reflective of an emphasis on physical competency, directly contradict and can be considered disruptive to the foundational integrity of PL. These lead to a narrow and dualistic understanding and operationalization of the concept (54).

Another approach highlighting an unintentional ableist view of PL development is the use of pedagogy grounded in unidirectional and linear processes [i.e., Long-Term Athlete Development Model, (55)]; specifically, PL education and practice associated with using a readiness model of motor development as a guide for instructional purposes (21). Within these practices, a substantive emphasis is placed upon the homogenous acquisition of motor skills as a predecessor to ongoing activity engagement (54, 56), while motivations related to participation are considered as afterthoughts. In other words, emphasis on physical development is prioritized, while the conceptualized entry point to PL development [i.e., motivation; (5, 15)] is downplayed. Such emphasis on movement skill acquisition as an antecedent to later physical activity behaviour also increases the focus placed on outcome over process (6, 24, 57). This cause-and-effect-type relationship holds the potential to impede future opportunities for individuals experiencing disability; those that may find a love and passion for, and thus develop a commitment to long-term engagement in.

Finally, a means to which ableism is reproduced in practice is in the lack of authentic perspectives from those possessing lived experience (i.e., interpersonal ableism) being used to guide PL development for individuals experiencing disability as a whole. Arbour-Nicitopoulos et al. (58) state that readily available and appropriately designed PL-enhancing programs for this population are essential for optimal life-long physical activity engagement. Yet, to date, little effort has been made to incorporate the suggestions of these individuals into programming (59). According to Durden-Myers and colleagues (13), an authentic perspective in the form of lived experience is essential to establish a meaningful connection to the physical activity context. Without its incorporation, there is increased potential to miss the mark concerning the intentional programming required to meet individualized needs, thus minimizing optimized experiences and increasing those perceived as unfavourable and fostering a disinclination to participate (60). It is this intentionality that is essential for fostering a PL context that embodies the true meaning of inclusion, affording opportunities for all to flourish (17, 18, 61).

In addition to original and authentic programming, the PL resources intended to support practitioner efforts to include those with less-privileged capabilities are of less quality. For example, resource development occurs outside the context being used and by those without the context-specific knowledge required to meet specific needs (24). These approaches highlight a skill set of typically developing individuals and an instructional approach that lessens individualized functional strengths, weaknesses, capabilities, and preferences. Furthermore, these approaches negate the diversity that enables individual uniqueness and potentially even the nature of inclusive, ethical practices (62). As described in Pushkarenko, Causgrove Dunn, and Goodwin (63), PL practices that fail to recognize diversity heighten the potential for negative experiences and trauma on a grander scale.

Another example of this lack of authenticity occurs through the limitations of PL assessment tools for those with diverse backgrounds [i.e., individuals experiencing disability; (64)]. Although debate continues as to the validity of assessment as a whole (65, 66), the increased emphasis on physical and skill-oriented behaviour indicates that there is more value toward identifying deficits in motor ability (57, 63, 67), thus reinforcing ableist ideals and limiting fidelity to the holistic underpinning of PL. Such focus leads to misconceptions regarding ability level, creating a sense of inferiority amongst those with less-privileged capabilities and act as a demotivating factor for continued physical activity engagement (68).

### Discussion and recommendations for practice

Considering the way that PL has been largely operationalized to date, there are numerous recommendations that practitioners can utilize as a means to ensuring equitable and inclusive practice, thus optimizing PL development opportunities for all. Although these recommendations are not an exhaustive list of possibilities, originating from an adaptive physical activity perspective and beyond that of individuals possessing lived experience, they are thought to represent a starting point to bridge the gap between PL knowledge and practice, and the facilitation of meaningful experiences of all, regardless of ability level.

## Adjusting the pedagogical approach

According to the United Nations Educational Scientific and Cultural Organization (69), literacy is not generic. Therefore, approaches to fostering its development require diversified strategies utilizing materials and information suitable to the learner's unique circumstances. Advocates of PL development reiterate these sentiments, suggesting that facilitating the unique PL journey of each learner requires an informed, pedagogical approach sensitive to the learner's needs (15, 70). In approaching PL development through such “pedagogical sensitivity,” those who facilitate physical activity experiences increase the likelihood of acting thoughtfully in the learning context, develop an individualized understanding of each learner, and grow increasingly flexible in their pedagogical position (70).

Pedagogical sensitivity is a tool for fostering PL development, moving gradually from a position of comfortability to one of increased complexity and challenge. A connection is built by initially establishing a relationship with learners, enabling learning to take place. Utilizing participant interests and fostering challenges considered non-threatening and comfortable for the individual illustrate this connection. Once established, pedagogues can begin to challenge learners, encouraging them to use their negative feelings as entry points to a deeper understanding of their learning experiences. Moreover, pedagogues afford assistance in developing confidence through listening and creating an environment where learners are poised to explore new situations, thereby broadening attitudes and abilities, interests, and a love of learning (i.e., engagement in purposeful physical pursuits).

The use of these skills individually, and the approach of pedagogical sensitivity as a whole, is not designated to any particular setting, therefore, is not the responsibility of any individual. As PL development occurs across environments [i.e., gymnasium, therapy, home, community; (71, 72)], a practice whereby pedagogical strategies are shared has been highly encouraged (73, 74). Through such means, consistent and continual support can be provided to the learner, thus optimizing their ongoing and unique PL journey by making the number of PL-enriched opportunities more achievable (75).

An example of pedagogical sensitivity in practice is witnessed when a PL facilitator demonstrates an awareness and responsiveness to the diverse needs, abilities, and interests of their students. For instance, during a basketball activity, there is a proactive consideration of the varying needs of all participants. Working collaboratively alongside participants, activity variations (i.e., adjustments to activities that are established prior to activity beginning, and those afford the opportunity to choose how one wants to engage) are designed to provide appropriate challenges and support for each individual based on level of ability. These may include variations to equipment size/height, varying distances, engaging in tasks independently or within a group, and/or simplifying or eliminating rules. This ensures that all students are actively engaged via the “happy medium” between challenge and success. The educator carefully observes and analyzes engagement, while simultaneously offering constructive feedback and questioning, tailored to participant's specific needs. They also foster a positive and inclusive learning environment, encouraging teamwork, respect, and collaboration amongst all involved. By being sensitive to the unique characteristics of their students and adapting their pedagogical approach accordingly, the facilitator promotes meaningful learning experiences and facilitates the development of PL for all learners.

Aligning with an approach that emphasizes pedagogical sensitivity are those which involve non-linear pedagogies (76, 77). A non-linear pedagogy prioritizes the learner, enables learners to learn through individual exploration, and facilitates holistic development of PL. Embracing the idea that learners are active participants in their own learning process, learners are given autonomy to design their learning content (76) and afforded the opportunity to explore, discover and problem solve [i.e., self-directed learning; (77)]. Claudia (76) further explained that the use of non-linear pedagogies positively influenced children's PL, self-determination, motor competence, self-efficacy, and overall physical activity engagement through adaptable, creative and self-directed movement experiences. This person-centred approach (78) has been positioned as a means of optimizing PL development and meaningful connections with the environment (58), empowering the learner “to guide instruction rather than being a pure recipient of it” (21).

In the context of recreation, a practical example of non-linear pedagogy can be seen in the organization and facilitation of a hiking excursion. Here, the recreation leader embraces a non-linear approach by allowing the participants to actively engage in decision-making and problem-solving throughout the hike. The leader encourages the group to collectively assess the environmental conditions, consider individual preferences and abilities, and collaboratively select the route or trail to explore that correspond with those preferences and abilities. This approach enables participants to adapt their hiking strategies based on changing circumstances, fostering critical thinking, communication, and navigation skills. The recreation leader serves as a facilitator, guiding discussions and offering support (if and when necessary), while allowing the participants to take ownership of their recreational experience. Through this pedagogical approach, participants have the opportunity to develop a sense of autonomy, problem-solving abilities, and a deeper connection with nature during the hiking excursion.

Affiliated with a pedagogically sensitive approach to PL development, and branching out from non-linear and constraints-based (see ecological dynamics below) approaches, Houser and Kriellaars (20) have recently advocated for PL-enriched pedagogy as a means to ensuring holistic development and meaningful experiences across a broad base of movement contexts. PL-enriched pedagogy is an intentional, person-centred design of physical activity opportunities, using that support the PL development of all students, both holistically (e.g., physical, psychological, social and creative) and inclusively [i.e., through the creation of individual agency; (20)]. Within this pedagogical approach, experiences are constructed through the use of strategies that deviate from a “technical movement focus” (20) to those that empower and create meaning on behalf of each individual learner—that which contribute to the motivation for continued engagement (8). As such, the PL facilitator and the learner work in tandem to facilitate a physical activity context that appropriately challenges each individual learner (79), and minimizes the negative emotions (i.e., fear, anxiety or self-doubt) that lead to negative movement experiences (20, 79).

In an inclusive physical activity context, a practical example of PL-enriched pedagogy can be observed in a community-based dance program for individuals of all ability levels. The dance instructor adopts an inclusive approach, creating a safe and supportive environment where all participants can engage in movement exploration and expression. The instructor provides initial direction to activities, while simultaneously empowering participants, allowing for individual adaptation and expression. For instance, modifications and variations to accommodate the diverse needs and abilities of participants, such as providing visual cues (i.e., pictures, videos), offering alternative movement options (i.e., seated, standing, lying down), or using assistive devices are utilized (i.e., chairs, walkers, canes). Additionally, the instructor encourages peer collaboration and support, fostering a sense of agency, community and acceptance among participants. Through this PL-enriched pedagogical approach, participants not only develop their movement ability but also gain confidence, self-expression, and a positive attitude towards physical activity. The focus is on individualized progress and enjoyment, ensuring that everyone feels included and valued.

## Attention to context

In response to PL development approaches that often overlook the diverse interests, abilities, and needs of individuals experiencing disability, Pushkarenko et al. (17) propose that PL-based programs should incorporate intentionality, addressing the unique requirements of each individual and recognize the influence of context in promoting success (48). As such, it is crucial to pay ample attention to the context in which individuals participate, as PL development thrives in settings that are perceived as motivating and enjoyable by participants (80). One approach to PL development that embraces the concept and emphasizes context is that of “ecological dynamics” (77, 81).

Based on ecological psychology (82) and dynamical systems theory (83), ecological dynamics places significant importance on PL as a personal journey of growth through individual experiences within diverse contexts (77, 84). It prioritizes the dynamic relationship between individuals and their environment, recognizing their continuous interaction (81). This approach provides a comprehensive understanding of PL and its development by highlighting the functional relationships among individuals, their environment, and the various constraints (such as physical, environmental, individual, and task-related) that influence movement experiences (81, 84, 85). It represents a shift in thinking from reductionist approaches to PL that focus solely on motor skill acquisition to one that emphasizes the perception of affordances (e.g., objects, places, surfaces, events, other people that provide opportunity and invite action) en route to establishing an optimal “individual-environment fit” (81). Within this framework, individuals develop motivation, confidence, and movement abilities to actively participate in activities while learning to adapt their skills in different contexts (81, 86).

By incorporating ecological dynamics into practice, PL facilitators can create enriching and playful learning environments that foster the development and adaptability of PL (77, 86). Through the manipulation of constraints, learners are encouraged to engage in exploration, problem-solving, and self-regulation (77), leading to the development of personalized movement solutions that align with their abilities, as well as the demands of the task and environment (86). Learners are empowered to take an active role in discovery and decision-making (86), enabling them to cultivate a deep understanding of movement dynamics, enhance adaptability and decision-making skills, and foster a lifelong engagement in physical activity (85, 87). This approach is increasingly recognized as individualized and aligned with the embodied experience, aligning more closely with the underlying philosophy of PL (75, 77).

A practical example of ecological dynamics in action can be observed during a cooperative movement activity. The PL facilitator designs an activity that incorporates diverse environmental and task constraints, taking into account the abilities and needs of all students. For instance, the teacher sets up stations with different types of equipment, such as balance beams, soft mats, and adaptive equipment like modified balls or tactile markers. The students, inclusive of those experiencing disability, are encouraged to explore the various movement possibilities and find creative solutions within their own abilities. The teacher fosters a supportive environment where students collaborate, assist one another, and adapt their movements to overcome challenges. By incorporating ecological dynamics principles, such as perceiving affordances, self-organization, and adapting to environmental constraints, participants experiencing disability can actively participate and contribute in meaningful ways, fostering their movement capacities, social integration, and overall PL. The teacher provides personalized support and modifications as needed, ensuring that every student can engage and benefit from the ecological dynamics-based learning experience within the environment.

## Developing communities of practice

In order to foster the development of PL in all individuals, it is crucial to involve all relevant stakeholders in the design, implementation, and delivery of PL-based programs. Belton and colleagues (88) emphasize the importance of engaging and understanding the perspectives of stakeholders, as it ensures “the relevance, ownership, and commitment to PL operationalization” (501). Adopting a “community of practice” approach allows for learning to be seen as a process of social transformation (89), moving away from a top-down dissemination of knowledge and fostering a dynamic and collaborative environment for knowledge creation. Here, active contribution and ongoing dialogue are encouraged, providing all stakeholders with the opportunity to challenge the misconception that participants must passively receive knowledge (90). In other words, stakeholders are empowered to actively engage, share their insights, and participate in meaningful discussions.

Given the dynamic and personalized nature of PL, practitioners must acknowledge and incorporate the authentic perspectives of learners to better understand their specific needs, desires, and values (21). Pushkarenko and colleagues (22) specifically explored the application of a community of practice approach with individuals experiencing disabilities and found that participants expressed a stronger connection to their environment, and collective efforts created a greater sense of community and belonging. By involving all stakeholders, including individuals experiencing disability as active contributors, it acknowledges their expertise and ensures equal participation in decision-making processes (62).

Moreover, collaboration among guardians, peers, teachers, therapists, and other stakeholders goes beyond specific contexts, allowing for PL development to occur in all aspects of an individual's life, challenging the notion that PL can only be cultivated under certain conditions (28, 91). The perspectives of these stakeholders hold significant value, as they bring lived experiences and a depth of knowledge that can inform a tailored approach to PL. The collective understanding of the specific barriers and facilitators related to ongoing physical activity engagement for individuals experiencing disability [see Ref. (92) for a comprehensive list], provides a context that enhances our comprehension of PL, ultimately contributing to the creation of “a broader community of movement pedagogues” (74). Yi and colleagues (91) liken this collaborative approach to a community of practice, advocating for the integration of diverse stakeholders and community partners to optimize physical activity experiences for everyone (28), ultimately advancing the shared objective of providing PL development opportunities for all.

## Enjoyment as an entry point to PL development

Research acknowledges the connection between an individual's motivation to engage in physical activity and the value they place on participation (92–94). Crucially, enjoyment plays a pivotal role in this relationship, as positive and enjoyable experiences, particularly those that empower and provide a sense of agency, foster a desire to engage in physical activity and cultivate intrinsic motivation for sustained involvement (22, 95). In the process of PL development, nurturing this intrinsic motivation becomes a pivotal starting point for establishing long-term commitment to physical activity (15). This intrinsic drive allows individuals to explore their capabilities, push their boundaries, and unleash their full embodied potential (8, 15, 22).

A noteworthy insight from Kwan and colleagues (96) is that enjoyment is a critical factor for success regardless of the type of program facilitating PL, as it influences feelings of confidence, physical competence, and ongoing motivation for participation across various movement contexts (92, 96). Aligning with Durden-Myers and colleagues' perspective on human flourishing and PL (61), positive engagement in physical activities nurtures individual goods and virtues, significantly contributing to the development of one's human-embodied potential (61). The concept of human flourishing is attainable by all, focusing on maximizing individual potential, regardless of inherent capabilities (61). To promote human flourishing and PL development in all individuals, practitioners should offer a diverse array of physical activity opportunities and allow participants to choose activities based on what they find enjoyable. By doing so, practitioners enhance the likelihood of participants remaining motivated to continue their engagement, thus creating an ongoing opportunity for PL development.

## Avoiding standardized assessment

Despite the widespread use of PL across various contexts, including education, sport, recreation, and public health (2–4), there remains an ongoing debate about its defining elements and practical implementation (7, 66). Efforts to measure and assess individual PL development have been made to enhance its applicability and accessibility (24). However, there is often an excessive focus on the physical domain, neglecting the other essential domains of PL, such as affective and cognitive (23, 24, 97). Furthermore, common assessment approaches are typically designed and administered by individuals without disabilities, which leads to assessments that prioritize standardization and performance, excluding those with diverse experiences of the world (49). Adopting an assessment-centric view of PL aims to define the competencies needed to be considered “physically literate,” overlooking the fact that each individual follows a unique PL journey (15). As PL is described as a dynamic and non-linear phenomenon, standardized and linear measurement systems are inadequate for capturing its complexity (98).

In line with the belief that PL is not a static achievement but an evolving process, a prominent suggestion for monitoring PL development is to chart one's individual journey over time (98, 99). However, it is crucial to acknowledge that each learner progresses through movements and physical activity contexts differently, making direct comparisons between individuals nearly impossible (98). Instead, the focus should be on how each individual optimizes their movement potential within their specific environment. Learners should play an active role in this process, actively involved in identifying their unique needs, desires, and assessment goals (61).

Any method used to document one's PL journey must be tailored to the learner's individual needs and consider all aspects of PL equally. This approach provides insights into and celebrates their participation in physical activities while offering feedback to inform future planning for lifelong engagement. By recognizing the dynamic nature of PL and embracing individuality, we can better support learners in their personal development and appreciation of physical activity.

## Reflexive engagement

To promote inclusive and meaningful physical activity opportunities for all individuals, it is crucial to carefully consider the ableist assumptions currently integrated into practice when developing physical activity programs. Acknowledging these assumptions and their impact is necessary to avoid the implementation of nonconscious, taken-for-granted practices that depreciate and devalue specific capabilities over others (62, 100, 101) and maintain an ethical commitment to pedagogy (62, 102, 103). By engaging in the process of reflexion, where practitioners consistently and critically question themselves and their actions (i.e., self-reflexivity), they may bring a level of introspection to their understandings, assumptions, and practices, increasing their awareness of what is right and wrong or what is suitable and not suitable within the environment in which they are attempting to foster meaningful experiences. This type of engagement invites new interpretations and conclusions, generating alternatives to traditional practices of PL development (103).

### Final thoughts

The intention of this paper has not been to pass judgment on current physical activity practices used to facilitate PL development, but to shed light on how these practices are only suitable for some. Despite being positioned as a concept applicable to all individuals regardless of ability level, PL is commonly understood through a lens of ableism and operationalized as a privilege to some and not others—perhaps unintentionally more so than anything else. As such, numerous practical considerations have been articulated, providing practitioners with opportunities to afford meaningful experiences for all, leading to life-long physical activity engagement. Recognizing that a change in practice will take time, the belief exists that practitioners can forego the immediate reinforcement and reproduction of ableism that individuals experiencing disability endure and reframe the PL narrative by considering such recommendations, thus holding fast to the true nature of inclusiveness and the underpinnings of PL as a whole.

## Data Availability

This article includes original contributions from the authors. Further inquiries can be directed to the corresponding author.

## Footnotes

^1^In this paper, we have adopted Whitehead's conceptualization of PL, as it aligns with the prevailing viewpoints shared by scholars who draw inspiration from Whitehead's perspective (1, 6, 7).

^2^The term “experiencing disability” is used throughout the document as it acknowledges the variation of “embodied sensations, social structures, and identities” that may be related to personal experiences of disability (16).

^3^The dominant narrative of PL privileges physicality in the form of acquiring a set of motoric skills considered to promote excellence and ongoing participation in physical activity (23, 24). This “body-as-machine” (25) operationalization represents an uncoupling of PL from its core meaning fostering development from the aspect of physical competence (6), rather than emphasizing something that is unique, relational, and holistic [i.e., Whiteheadian conception of PL; (5, 15)].
